# One-stage reconstruction of extensive exposed tibia on malnourished patient using single-layer Integra and amino acid supplements: A case report and literature review

**DOI:** 10.1097/MD.0000000000037098

**Published:** 2023-02-02

**Authors:** Kuan-I Lee, Yun-Nan Lin

**Affiliations:** a School of Medicine, Kaohsiung Medical University, Kaohsiung, Taiwan (R.O.C.); b Division of Plastic Surgery, Department of Surgery, Kaohsiung Medical University Hospital, Kaohsiung Medical University, Kaohsiung, Taiwan (R.O.C.); c School of Post-Baccalaureate Medicine, College of Medicine, Kaohsiung Medical University, Kaohsiung, Taiwan (R.O.C.)

**Keywords:** artificial dermis, exposed tibia, one-stage reconstruction, skin grafts, traumatic injuries

## Abstract

**Rationale:**

Extremity injuries resulting from motor vehicle collisions, especially those leading to bone-exposed wounds, present challenges for achieving effective wound coverage. Such injuries are susceptible to complications including infections, osteomyelitis, and unexpected amputations due to inadequate blood supply. Severe traumatic degloving injuries often entail damage to the surrounding blood vessels, making local or free flaps impractical choices in many cases. Consequently, treatment options may vary based on distinct clinical scenarios, with no standardized guidelines available. Our study introduces an integrated approach utilizing dermal substitutes and skin grafts as a safer treatment modality for managing large-area tibial exposure resulting from traffic accidents.

**Patient concerns:**

A 66-year-old male with a compromised nutritional status was struck by a car while riding a motorcycle. Previous attempts using double-layer Integra and negative pressure wound therapy (NPWT) for two-stage reconstruction have been unsuccessful.

**Diagnoses:**

Computed tomography imaging studies revealed multiple comminuted and displaced fractures involving the left femoral shaft, left proximal tibia, left patella, and proximal fibula, as well as a fracture of the right fibular shaft and an avulsion fracture of the right distal medial femur. The patient’s condition corresponded to Type 3B in the Gustilo classification for open fractures, and the patient had an Injury Severity Score of 25.

**Interventions:**

We applied a one-stage reconstruction involving single-layer Integra, split-thickness skin grafts, NPWT, and nutritional supplements containing various amino acids.

**Outcomes:**

By implementing an integrated treatment approach and providing diligent wound care over a total of 2 months, the patient achieved successful healing and expressed satisfaction with the postoperative results.

**Lessons:**

This study offers insights into the effectiveness of employing one-stage reconstruction for traumatic injuries with extensive exposed tibias. In addition, it underscores the impact of a patient’s nutritional status on wound healing and introduces a potential solution for similar challenging cases.

## 1. Introduction

Extremity injuries occur most often because of falls, work-related accidents, and motor vehicle collisions. In cases of severe trauma, degloving injuries leading to bone-exposed wounds present an additional layer of complexity for achieving adequate wound coverage. The exposed bone does not have sufficient blood supply, making it prone to several severe complications including infections, osteomyelitis, and unnecessary amputations.^[1]^ To avoid these complications, it is critical to effectively cover the area.^[2,3]^

The current management of soft tissue loss degloving injuries with bone-exposed wounds is multidisciplinary, including primary or direct closure, skin grafts, and local and free flaps.^[4]^ Considering that severe traumatic degloving injuries are usually accompanied by large-area tissue defects and surrounding harming vessels, local or free flaps are not always a viable option in such cases. In other words, flap reconstruction may be inappropriate for flap reconstruction.^[5]^

In this study, we present a case of a 66-year-old malnourished male who sustained a sizable tibia-exposed wound. Successful wound healing was achieved through the integration of nutritional supplements, negative pressure wound therapy (NPWT), and dermal substitutes, combined with skin grafts in a one-stage reconstruction.

## 2. Case Report

We present a case of a 66-year-old healthy male who was struck by a car while riding a motorcycle. Initial assessment revealed left leg deformity and complete paralysis in all 4 limbs, while the patient remained conscious and alert. The patient was immediately transported to our emergency department for medical intervention. Upon arrival, the medical team observed continuous bleeding and hypotension, necessitating urgent blood transfusion and vasopressor administration. Computed tomography imaging studies indicated a right tentorial subdural hemorrhage and multiple comminuted and displaced fractures involving the left femoral shaft, left proximal tibia, left patella, and proximal fibula, as well as a fracture of the right fibular shaft and an avulsion fracture of the right distal medial femur. The patient’s condition corresponded to Type 3B in the Gustilo classification of open fractures. The patient had an injury severity score of 25 and was diagnosed with a major trauma.

**Figure 1.**
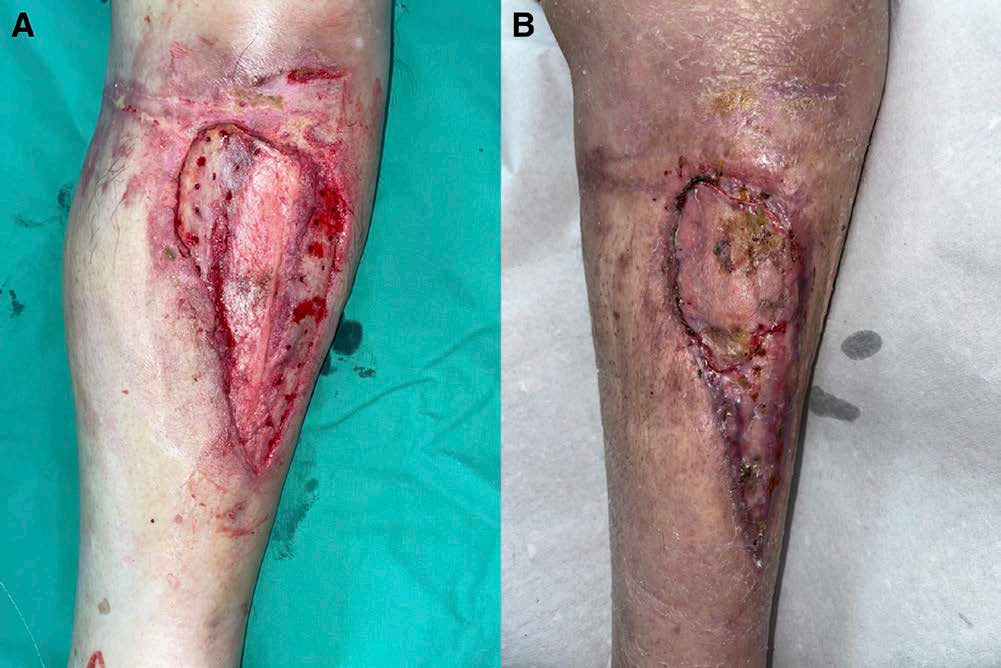
Images before and after extensive tibia wound treatment. (A) Initial state featuring a 54.65 cm^2^ avulsion wound and an 18.62 cm^2^ exposed tibia area. (B) Successful healing achieved after a 2-month integrated treatment regimen and meticulous wound care.

Owing to the patient’s unstable vital signs, he was promptly transferred to the intensive care unit (ICU), where an orthopedist performed bedside wound debridement, suturing, and pin insertion for skeletal traction. Following a week of critical care in the ICU setting, a crush injury to the left lower leg with associated skin necrosis was identified. An orthopedic surgeon performed debridement, open reduction, and internal fixation of the femoral shaft fracture. A triangular avulsion wound, measured 54.65 cm^2^, was observed over the anteromedial aspect of the left lower leg. Notably, the exposed area of the tibia, measuring 18.62 cm^2^, accounted for > 34% of the total wound area (Fig. 1A). Extensive bone exposure was initially managed with NPWT. Subsequently, a plastic surgeon was consulted to provide advanced wound care to the patient.

**Figure 2.**
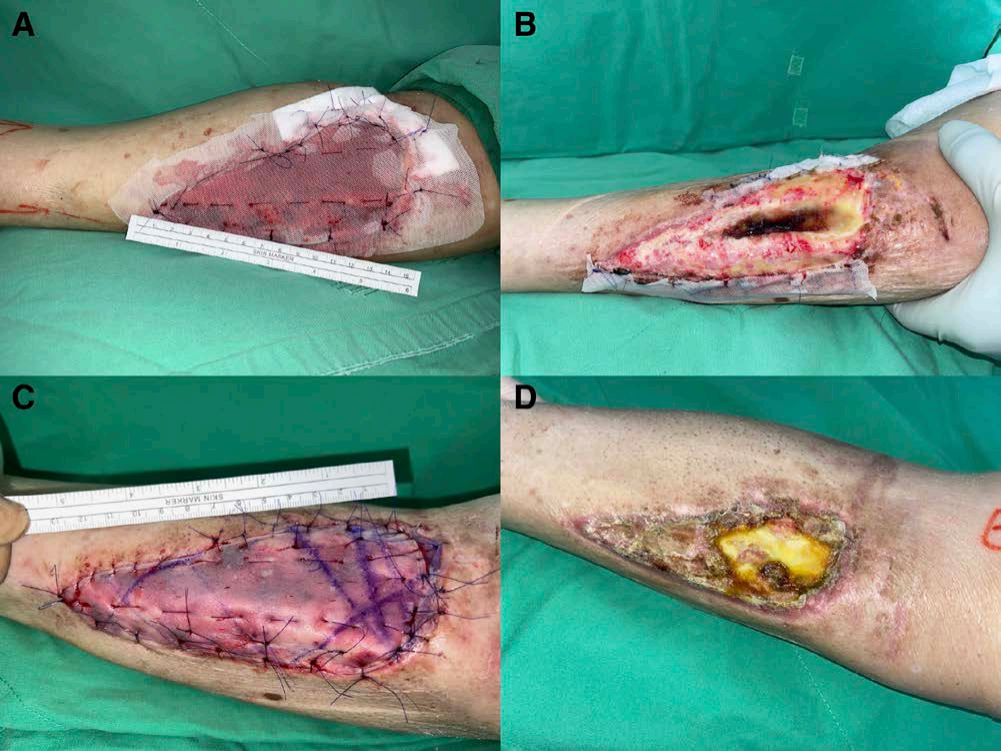
Treatment process for tibia-exposed wound. (A) Initiation phase involved the application of Integra and NPWT to the tibia-exposed wound region. (B) Granulation tissue growth was nearly absent during the 1st-week follow-up assessment. (C) Second treatment phase included one-stage reconstruction with the introduction of amino acid supplements, NPWT, single-layer Integra, and STSG. (D) Diligent wound care over 6 weeks resulted in successful healing of over 82% of the wound area.

In the initial phase of treatment, surgical debridement was performed to remove necrotic tissue. Considering the patient’s advanced age and comminuted fractures around the plateau area and proximal tibia, he was not an ideal candidate for bone fenestration and flap reconstruction. Although biochemical blood tests revealed a markedly low albumin level (2.6 g/dL), urgent wound healing was necessary. After explaining the situation to the patient, we applied NPWT and Integra dermal regeneration template (IDRT, Integra LifeSciences, Princeton, NJ), a type of dermal substitutes, to the exposed wound area of the tibia. This was performed with the aim of preparing a wound bed for future skin grafting. However, granulation tissue failed to grow (Fig. 2B). Considering the patient’s compromised nutritional status, a nutritional supplement containing L-glutamine, L-arginine, and calcium HMB, was introduced during the second phase of treatment, which was combined with NPWT, single-layer Integra, and 0.006 inch of split-thickness skin grafts (STSG) in a one-stage reconstruction (Fig. 2C).

**Figure 3.**
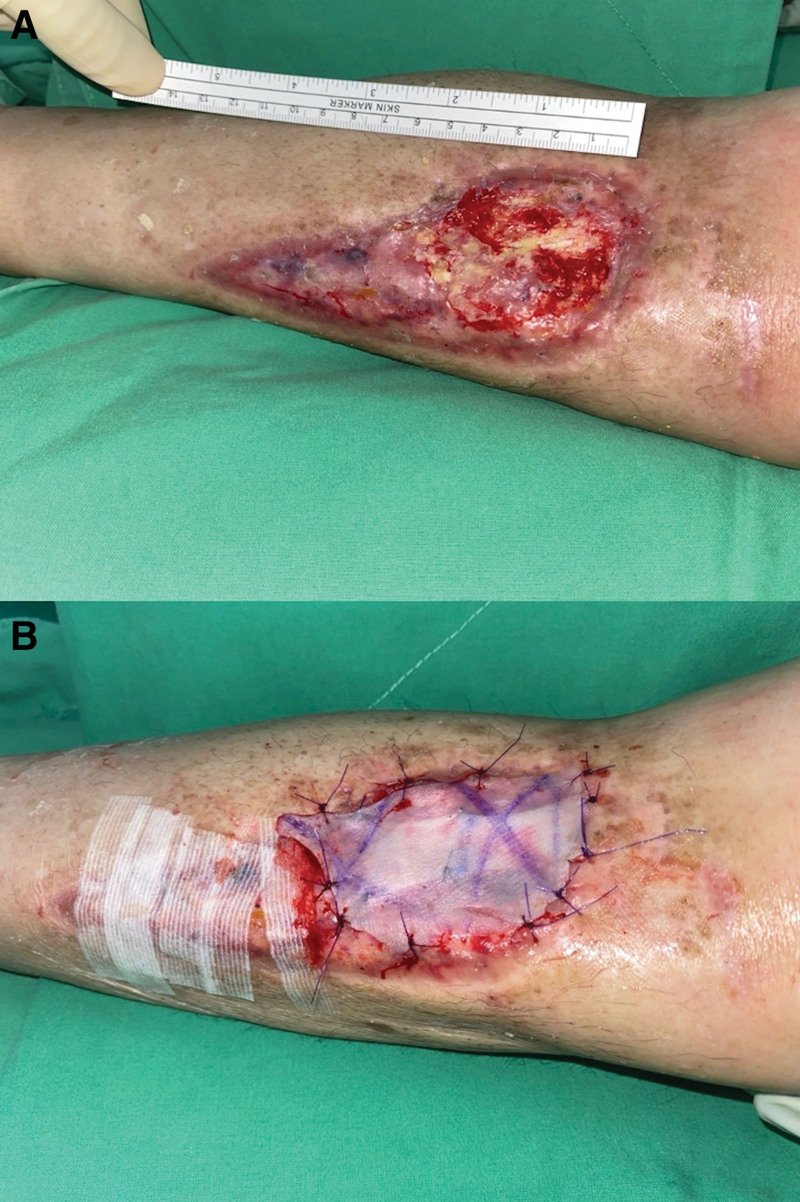
Secondary STSG were performed due to partial sloughing of skin grafts. (A) After adequate debridement, fair granulation tissue was observed over the tibia. (B) Secondary STSG procedure performed due to suboptimal graft adherence.

After 6 weeks of diligent wound care, encouraging progress was noted, with the wound bed displaying granulation tissue and no adverse events and other unanticipated problems were observed. Over 82% of the wounds healed successfully (Fig. 2D). The residual area requiring treatment was 9.47 cm^2^ (Fig. 3A). A secondary STSG procedure was performed to address the areas where graft adherence was suboptimal (Fig. 3B). After a total of 2 months of meticulous wound management, the extensive tibial exposed wound achieved complete healing (Fig. 1B). The patient expressed satisfaction with both the aesthetic appearance of the resulting scar and the postoperative outcomes during latest follow-up. Written informed consent was obtained from the patient for the publication of this case report and accompanying photographs.

## 3. Discussion

Reconstructive surgery plays an important role in the closure of severely traumatic wounds. However, traumatic wounds with exposed bones or tendons continue to present challenges for surgeons. Over the years, extensive research has been conducted to determine the best way to treat complicated wounds.^[6–9]^ While free flaps have shown excellent results in head and neck reconstruction, their success rate in lower-limb reconstruction is typically lower.^[10]^ According to Godina,^[11]^ free flap reconstruction carries a higher complication rate, with a risk of flap loss in post-traumatic cases. These complications are mostly attributed to refractory spasms of recipient vessels, a condition known as post-traumatic vessel disease.^[12]^ Parrett et al^[13]^ discussed a current shift in the treatment approach for traumatic wounds with exposed bones. In the past, free flaps were commonly used for reconstruction, but there has been a trend toward simpler methods such as local flaps and skin grafts. Skin grafts, often combined with dermal substitutes^[8,9,14–17]^ or muscle flaps^[18–21]^ have been reported in several studies. These shifts have been attributed to the early involvement of plastic surgeons^[22]^ as a result of a reduced risk of complications.

Rios-Luna et al^[23]^ demonstrated the basic principles of exposed tibial reconstruction using local flaps. The gastrocnemius muscle flap was proposed for the proximal third of the tibia, whereas the soleus muscle flap could be used to reconstruct the middle third of the tibia. The sural flap and posterior tibial perforator flap covered the distal third of the tibia. Reverse sural flaps and bipedicled fasciocutaneous flaps have also been mentioned in a few studies as successful options for reconstructing distal wounds.^[24–26]^ Tibialis anterior muscle flaps have been reported to yield acceptable outcomes in smaller defects, especially in those with exposed bone over the middle third of the leg.^[20,27,28]^

Previous studies have also indicated that combining IDRT, a dermal substitute, with NPWT therapy is a safe and effective method for preparing the wound bed for future skin grafting reconstruction in bone-exposed wounds.^[29–31]^ Skin grafts, according to the reconstructive ladder, maybe the simplest method for managing complicated wounds, with the lowest complication rate.^[32]^ However, achieving successful healing of bone-exposed wounds with skin grafts remains a formidable challenge.^[33]^ IDRT appears to offer a promising strategy with the potential to bridge this gap, as it facilitates wound preparation for subsequent skin grafting through a 4-step process involving imbibition, fibroblast migration, neovascularization, and remodeling and maturation.^[34]^

In our patient, the initial attempt to utilize a combination of IDRT and NPWT to encourage wound bed granulation was unsuccessful. This outcome may be attributed to the patient’s advanced age and compromised nutritional status. Recent systematic reviews have highlighted the potential positive effects of arginine or glutamine on the wound healing process.^[35,36]^ Therefore, we incorporated amino acid nutritional supplements into the treatment regimen during the second attempt to address this complex wound. Furthermore, we also attempted to reconstruct the bone-exposed defect with IDRT, covered immediately with meshed STSG, without waiting for visible signs of tissue granulation. This integrated strategy culminated in successful healing of the patient’s wound over a total of 2 months. It is worth mentioning that bone fenestration was not used in our patient due to the orthopedic surgeon’s recommendation for future intramedullary nailing fixation. Following multiple comminuted fractures, the bone fenestration technique may render the bone even more fragile and unstable. This situation makes reconstruction more complicated and difficult in this patient.

**Figure 4.**
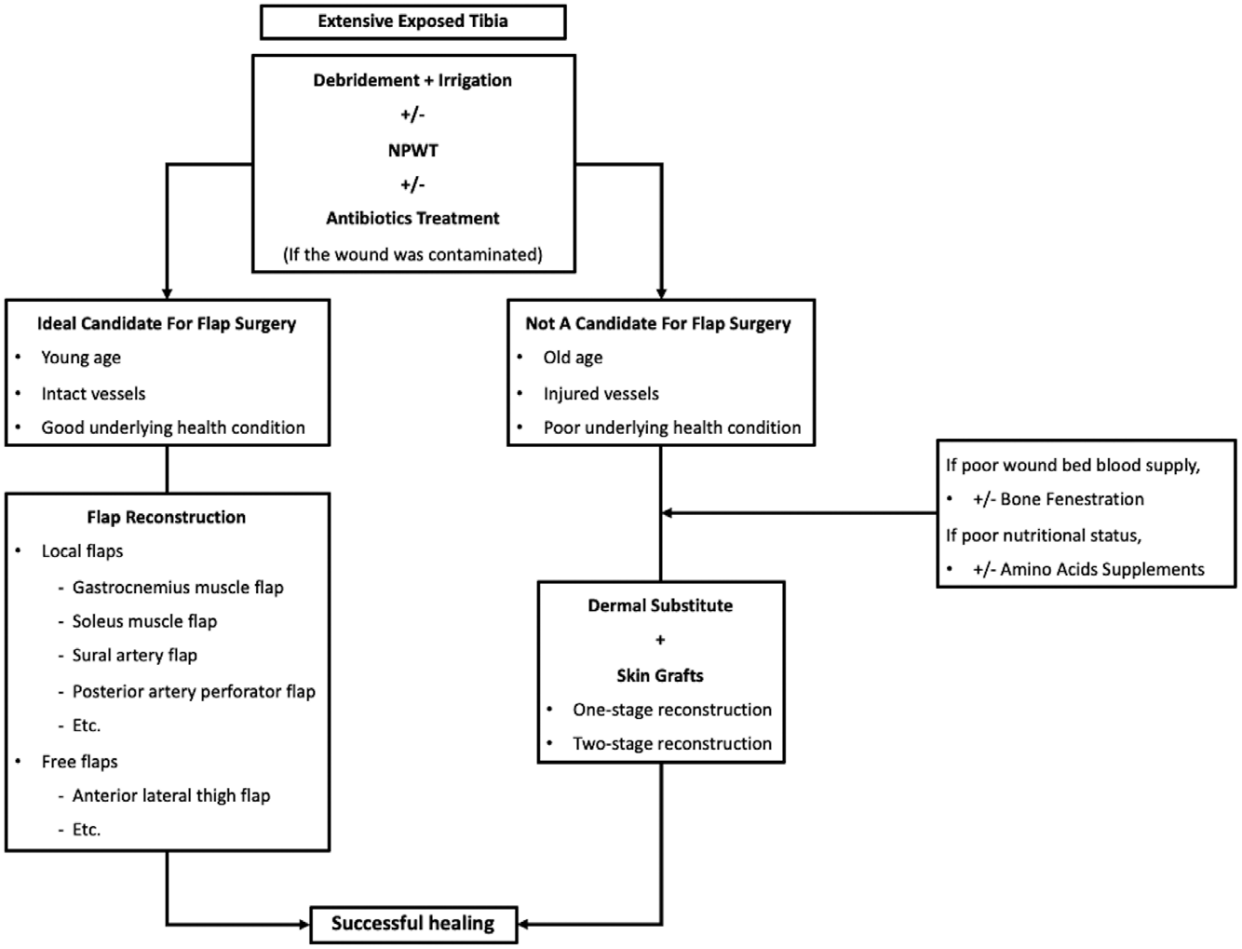
Flow chart showing strategic approach for the management of extensive exposed tibia. Initially, we recommend debridement and irrigation. The use of NPWT and antibiotics depends on the clinical scenarios. If the patient is an ideal candidate for flap reconstruction, we can choose between local or free flaps. However, local flaps are generally considered a safer treatment option due to their lower complication rates. For elderly patients, individuals in poor health, or those with injured vessels, combining dermal substitutes with skin grafts may offer an alternative and promising treatment strategy.

Papa et al^[37]^ reported that both double-layer and single-layer dermal substitutes show identical three-dimensional structures in histological studies conducted after application. However, the adoption of single-layer Integra coupled with immediate skin grafting presents distinct advantages, sparing the need for 2 separate surgical interventions. Successful one-stage reconstruction of soft tissue defects using IDRT has been reported in several studies. Koenen et al^[38]^ and Demiri et al^[39]^ demonstrated successful one-stage reconstruction using single-layer Integra® to treat facial and upper extremity skin defects. It is worth noting that the above cases had no exposed bone areas. Only Kosutic et al^[40]^ reported the use of one-stage reconstruction using single-layer Integra on post-burn bone-exposed scalp defects measuring 10 × 6 cm and 6 × 4 cm. Our study demonstrated the potential of this technique by focusing on its application to extensive bone-exposed wounds over the extremities, a previously unexplored application. We provide a summary of the treatment approaches for extensively exposed tibia using flap reconstruction and dermal substitutes (Tables 1 and 2). Considering the similarity in treatment strategies, we included cases caused by trauma and burns. In this study, we proposed a treatment algorithm (Fig. 4). More clinical experience is needed to provide a higher level of evidence for developing a strategy for treating extensively exposed tibial wounds.

**Table 1:** Summary of previously reported treatments of severe exposed tibia with flap reconstruction.

Authors (publication year)	Etiology	Case	Age, year	Total wound area, cm^2^	Tibia bone area, cm^2^	Treatment	Time to healing (days)	Outcomes	Remarks
Hallock (2002)^[27]^	Traffic accidents (2) Fall (1)	3	6–82	20–50	NA	Gastrocnemius muscle flap + tibialis anterior muscle flap + skin grafts (1) Tibialis anterior muscle flap + skin grafts (2)	NA	Healed	Calf skin necrosis at site of medial gastrocnemius donor site was noted in one case.
Heymans et al (2002)^[41]^	Burns	2	45–60	NA	Case1: – Case2: 12	1st: Skin grafts -> failed 2nd: Adipofascial flap + skin grafts -> healed	NA	Healed	–
Kamath et al (2006)^[42]^	Traumatic injuries	10	23–48	9–35	NA	Perforator based flap	NA	Healed	A deep seated infection resulting in sinus formation needing further debridement was noted in one case.
Pu (2006)^[18]^	Vehicle accidents (4) Fall (2) Infected nonunion (2)	8	12–52	9–60	NA	Reversed hemisoleus muscle flap + skin grafts	NA	Healed	2 patients developed insignificant distal flap necrosis, and they were treated subsequently with debridement and flap readvancement. 1 patient lost part of the skin graft over the muscle flap and was simply treated with reskin grafting.
Darwish (2010)^[26]^	Chronic ulcers (3) Traumatic injuries (20)	23	11–55	15–75	NA	Fasciocutaneous bipedicled flap	NA	Healed	2 patients were observed partial necrosis of the flap, due to superficial sloughing of the lateral edge of the flap, which healed conservatively.
Elghamry (2011)^[43]^	Traumatic injuries	4	28–55	12–16	NA	Ankle extensor-tendon-sheath flap + skin grafts	120–180 days	Healed	–
Megahed (2011)^[28]^	Burns (7) Traumatic injuries (9)	16	14–67	NA	NA	Tibialis anterior muscle flap + skin grafts	NA	Healed	Partial flap loss occurred in one patient and wound dehiscence managed by secondary suture in another case were noted.
Contedini et al (2012)^[44]^	Crush trauma	1	32	360	NA	1st: Latissimus dorsi muscle flap -> failed 2nd: Cross-leg flap + skin grafts -> healed	NA	Healed	Latissimus dorsi muscular free flaps failed for vascular thrombosis.
Esezobor et al (2012)^[45]^	Motorcycle accident	1	22	276	NA	Reverse sural artery fascio cutaneous flap + hemisoleus muscle flap	NA	Healed	–
Krishnamoorthy et al (2013)^[19]^	Traffic accident	1	28	84	NA	Medial gastrocnemius flap + saphenous fasciocutaneous flap + medial hemisoleus flap + Skin grafts	NA	Healed	–
John et al (2015)^[46]^	Traumatic injuries	11	21–66	9–35	NA	Peroneal artery perforator flap	18–40 days	Healed	1 patient sustained partial flap necrosis, which was debrided, and another local flap was performed.
d’Avila et al (2014)^[7]^	Traumatic injuries (45)	45	9–84	NA	NA	Gastrocnemius flap and/or soleus flap + Skin grafts	NA	Healed	45 patients included in this study were tibia and/or fibula fractures.
Ince et al (2014)^[47]^	Car accidents (7) Gun shot (4)	11	21–57	NA	NA	Reverse-flow islanded sural flap	NA	Healed	Although the wound or bone-exposed area of the patients were not mentioned in this study, the biggest flap measured 16 × 11 cm and the smallest one, 5 × 6 cm. The longest pedicle was 27 cm long and the shortest one 21 cm. 1 patient had partial necrosis of the flap, and the necrosis healed secondarily.
Zhang et al (2015)^[6]^	Traffic accidents (20) Crush injuries (6) Amputation (1)	27	18–60	60–252	NA	Anterolateral thigh perforator flap	NA	Healed	5 patients suffered from partial skin necrosis at flap edge and 2 patients were observed flap loss caused by venous or arterial thrombosis. In long term follow-up, 3 patients had sinus formation and one patient presented reinfection at the sixth postoperative month.
Jaiswal and Pu (2015)^[20]^	Traumatic injuries	1	29	15	NA	Tibialis anterior muscle flap + skin grafts	NA	Healed	Another patient in this study focused on reconstruction of an exposed Achilles tendon wound, so it was not included in our table.
Andrea Dessy et al (2017)^[48]^	Run over by a trailer	1	39	NA	NA	1st: skin grafts -> failed 2nd: Propeller flap based on posterior tibial perforator + skin grafts -> Healed	NA	Healed	–
Yasuda et al (2017)^[21]^	Traffic accident	1	17	91	NA	Gastrocnemius muscle flap + soleus muscle flap + flexor hallucis longus flap + skin grafts	21 days	Healed	The distal third of the tibia can often not be covered with the gastrocnemius muscle and soleus muscle flaps. The potential of an additional proximally based flexor hallucis longus flap was reported in this study.
Yoshimatsu et al (2018)^[49]^	Motorcycle accident	1	29	500	NA	Deep inferior epigastric artery perforator free flap	NA	Healed	The flap was based on both bilateral DIEPs and on the superficial circumflex iliac artery (SCIA).
Hassanpour et al (2018)^[50]^	Traumatic injuries	12	22–58	NA	NA	Soleus muscle flap + sural artery fasciocutaneous flap	NA	Healed	1 patient developed insignificant distal flap necrosis who was treated subsequently with surgical debridement and flap readvancement.
Malahias et al (2020)^[51]^	Fall (3) Traffic accidents (2)	5	32–79	16–84	NA	Peroneus brevis muscle flap + skin grafts	63 days	Healed	5 patients out of 21 patients in this study involved with exposed tibia.

**Table 2:** Summary of previously reported treatments of severe exposed tibia with dermal substitutes and skin grafts.

Authors (publication year)	Etiology	Case	Age, year	Total wound area, cm^2^	Tibia bone area (cm^2^)	Treatment	Time to healing (days)	Outcomes	Remarks
Gáspár et al (2006)^[14^	Burns	1	72	NA	18	Dermal substitute (AlloDerm) + skin grafts	14 days	Healed	–
Ohara et al (2010)^[16]^	Burns	1	82	NA	NA	Dermal substitute + skin grafts	210 days	Healed	–
Kang et al (2010)^[15]^	Traumatic injuries	1	38	300	NA	NPWT + dermal grafts + Skin grafts	NA	Healed	–
Yeong et al (2012)^[17]^	Burns	4	28–60	–	6–200	Dermal substitute (Integra) + skin grafts	NA	Healed	4 out of ten patients in this study suffered from exposed tibia. 3 cases had a successful take of skin grafting on neodermis, and 1 case had partial skin graft loss due to infection.
Graham et al (2013)^[8]^	Traumatic injuries	5	40.1 ± 22.6 (mean age)	50–1000	NA	Dermal substitute (Integra) + Skin grafts	NA	4 healed 1 amputation	This study discussed the reconstruction of bone-exposed wound in lower extremities instead of exposed tibia specifically. 2 patients experienced clinically significant infections.
Verbelen et al (2016)^[2]^	Burns	1	44	Right leg: 50 Left leg: 40	NA	1st: Flap reconstruction -> failed 2nd: Dermal substitute (Integra) was applied, attempting to cover the exposed tibias -> failed 3rd: Flap reconstruction -> failed 4th: NPWT + bone fenestration + dermal substitute (Glyaderm) + skin grafts -> healed	42 days	Healed	–
Hughes et al (2021)^[9]^	Traumatic injuries	1	86	250	NA	Bone fenestration + dermal substitute (Integra) + skin grafts	56 days	Healed	–
Our case	Traumatic injury	1	66	55	19	1st: NPWT + dermal substitute (Integra) + skin grafts -> failed 2nd: NPWT + dermal substitute (Integra) + amino acids supplement + skin grafts -> healed	56 days	Healed	Two-stage reconstruction failed in the first attempt then shift to one- stage reconstruction.

We acknowledge the limitations of this study. First, the technique presented may not be universally applicable, particularly for larger bone-exposed soft tissue defects. Second, variations in patients’ nutritional status and the challenge of quantifying these states make it difficult to establish a definitive threshold for incorporating amino acid supplements into combination therapies. Finally, while our study offers promising outcomes, it is imperative to recognize that it is based on a single case. Further research encompassing case-control studies with a larger cohort is necessary to provide a more comprehensive understanding of the potential superiority of one-stage reconstruction over two-stage reconstruction in terms of healing potential, reliability, and reproducibility.

## 4. Conclusion

This study highlights the potential of one-stage reconstruction in patients with extensive bone exposure, which benefits patients by avoiding 2 separate surgical interventions. Our study also highlights how a patient’s nutritional status influences wound healing, and that amino acid supplements may be viable solutions to this challenge. Our experience provides valuable insights for future cases characterized by similarly severe conditions.

## Acknowledgments

We thank the patients who participated in this study.

## Author Contributions

**Writing – original draft:** Kuan-I Lee.

**Writing – review & editing:** Kuan-I Lee, Yun-Nan Lin.

**Conceptualization:** Yun-Nan Lin.

**Resources:** Yun-Nan Lin.

**Supervision**: Yun-Nan Lin.

**Visualization:** Yun-Nan Lin.
